# MidExDB: A database of *Drosophila *CNS midline cell gene expression

**DOI:** 10.1186/1471-213X-9-56

**Published:** 2009-11-10

**Authors:** Scott R Wheeler, Stephanie B Stagg, Stephen T Crews

**Affiliations:** 1 Program in Molecular Biology and Biophysics, Department of Biochemistry and Biophysics, The University of North Carolina at Chapel Hill, Chapel Hill, NC 27599-3280, USA

## Abstract

**Background:**

The *Drosophila *CNS midline cells are an excellent model system to study neuronal and glial development because of their diversity of cell types and the relative ease in identifying and studying the function of midline-expressed genes. In situ hybridization experiments generated a large dataset of midline gene expression patterns. To help synthesize these data and make them available to the scientific community, we developed a web-accessible database.

**Description:**

MidExDB (*Drosophila *CNS Midline Gene Expression Database) is comprised of images and data from our in situ hybridization experiments that examined midline gene expression. Multiple search tools are available to allow each type of data to be viewed and compared. Descriptions of each midline cell type and their development are included as background information.

**Conclusion:**

MidExDB integrates large-scale gene expression data with the ability to identify individual cell types providing the foundation for detailed genetic, molecular, and biochemical studies of CNS midline cell neuronal and glial development and function. This information has general relevance for the study of nervous system development in other organisms, and also provides insight into transcriptional regulation.

## Background

The neurons and glia that comprise the *Drosophila *CNS midline cells are an excellent model system to study neurogenesis and gliogenesis [1,2]. This is due to their highly recognizable location at the midline of the embryo, small number of cells, diversity of cell types, large number of identified genes and associated expression patterns, and the ability to identify individual cell types across embryonic development. In each ganglion, there are ~18 midline neurons including glutamatergic/octopaminergic motorneurons, peptidergic motorneurons, dopaminergic interneurons, and glutamatergic interneurons [3]. There are two molecularly distinct populations of midline glia (MG): the anterior MG (AMG) ensheath the commissural axons that cross the midline and the posterior MG (PMG) have unknown function. Study of the midline cells has been instrumental in studying programmed cell death, the role of the Single-minded (Sim) master regulatory transcription factor protein, neuronal and glial cell fate, neuron-glia interactions, and how diffusible factors control axon guidance. The insect midline cells strongly resemble the floorplate cells that reside at the midline of the vertebrate spinal cord [1]. Both the *Drosophila *midline cells and vertebrate floorplate cells are important embryonic signaling centers - in *Drosophila*, the midline cells are a source of signals responsible for axon commissure formation, muscle cell migration, and the formation of the ventral epidermis and mesodermal dorsal median cells.

**Figure 1:**
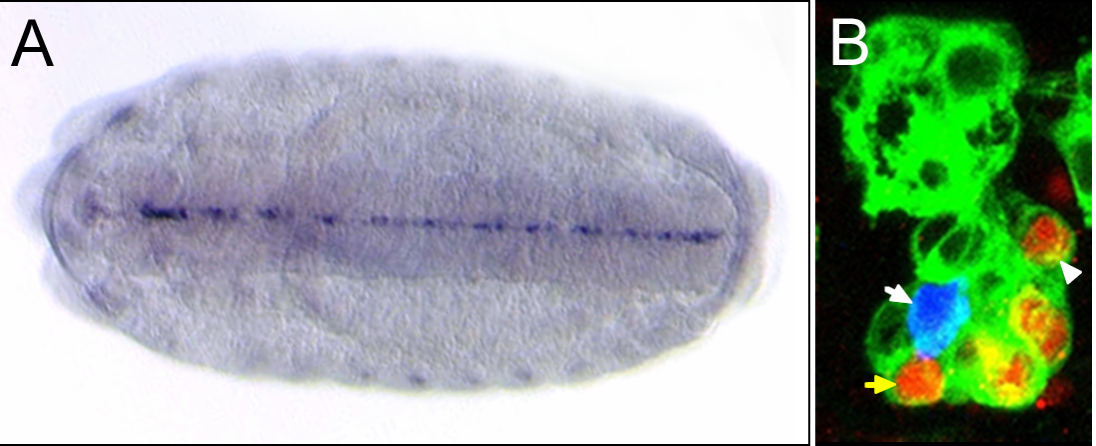
**CNS midline gene expression**. (A) In situ hybridization (AP histochemistry) of the *Poly-glutamine tract binding protein 1 *(*PQBP-1*) gene at stage 15 of embryonic development showing prominent midline cell expression. Ventral view; anterior left. (B) Sagittal view of a single segment of a *sim-Gal4 UAS-tau-GFP *embryo at stage 17, in which *tau-GFP *is expressed in all midline cells and facilitates midline cell identification by confocal microscopy. The embryo was immunostained with: (1) anti-GFP (green) to visualize the cytoplasm of all midline cells and (2) anti-Engrailed (red), which stains the nuclei of ventral unpaired median interneurons (iVUMs; yellow arrow points to 1 iVUM), median neuroblast (MNB), and MNB neuronal progeny (arrowhead points to 1 MNB progeny). The embryo was also hybridized to a probe for *pale *(blue) which stains the H-cell (white arrow). Dorsal top; anterior left.

While *Drosophila *midline cell gene expression has been studied for over 20 years, a major advance was a large-scale in situ hybridization screen, in which the midline expression patterns of 224 genes were identified and documented throughout embryonic development (Figures 1A) [2]. The genes analyzed were identified based on a variety of approaches, including enhancer trap screens, microarray experiments, the existing scientific literature, and in situ hybridization screens, including midline-expressed genes identified from the Berkeley Drosophila Genome Project (BDGP) embryonic in situ hybridization gene expression database [4]. These data are referred to as "AP data", since the in situ-hybridized embryos were stained using alkaline phosphatase (AP) histochemistry and imaged by differential interference contrast (DIC) microscopy. Subsequently, the expression of 77 genes was mapped at 5 stages of embryonic development using multi-label fluorescence confocal microscopy (Figure 1B) [3,5]. These fluorescent data are referred to as "confocal data". The confocal data provided the ability to: (1) identify individual midline cell types at all stages of embryonic development, (2) analyze how gene expression changes in individual cells during development, and (3) carry-out sophisticated genetic experiments for studying midline cell gene function and transcriptional circuitry. In addition, this work provided key insights allowing a refinement of how midline cells develop [5]. Consequently, to facilitate the ability of the scientific community to access and use both types of midline gene expression data, we created a web-based searchable database, MidExDB (*Drosophila *CNS Midline Gene Expression Database;  or accessible from the Crews Lab home page at ). MidExDB contains CNS midline cell gene expression data at both low-resolution (AP data) and high-resolution (confocal data).

## Construction and content

**Figure 2:**
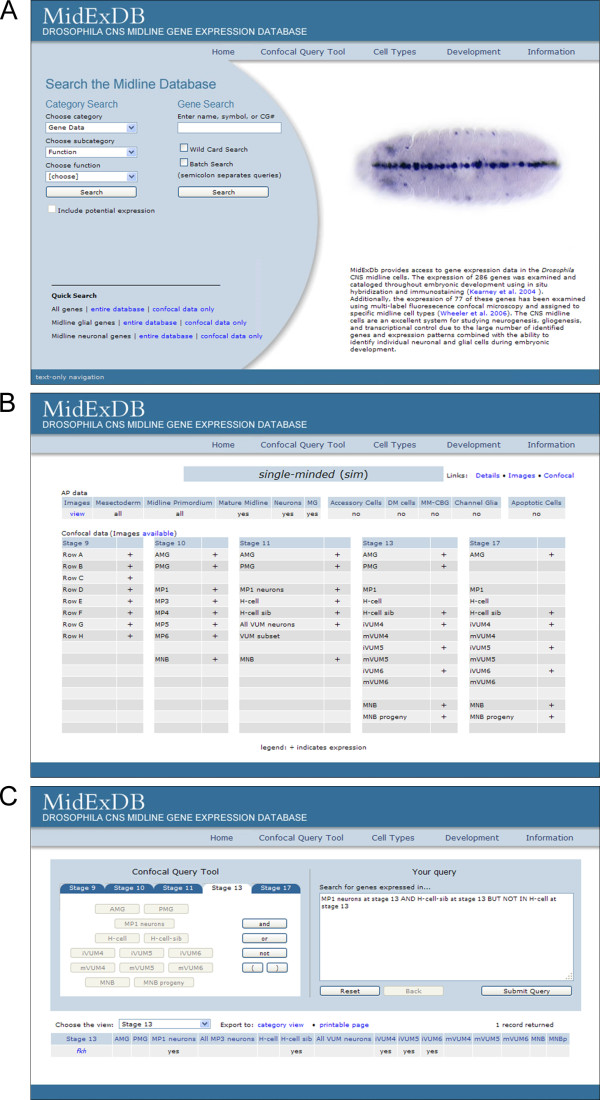
**MidExDB search modes**. (A) MidExDB Home page showing Category Search, Gene Search, and Quick Search. (B) Results of the Confocal view for the *sim *gene indicating its expression (+) at 5 stages of development. (C) Results from the Confocal Query Tool. The query is indicated on the right with the results listed below, showing that only the *fkh *gene matched the query.

MidExDB is a relational database created using Microsoft SQL server 2000 and Visual Studio. At the top of each page, there is a navigation bar with links and pull-down menus entitled: Home, Confocal Query Tool, Cell Types, Development, and Information (Figure 2). The Home page provides access to AP and Confocal data searches. The Confocal Query Tool allows the user to search for cell-type and stage-specific gene expression data. The Cell Type and Development menus provide links describing the individual midline cell types and summaries of developmental events. The Information menu includes links to Help, Protocols, References, the Crews Lab homepage, and email address.

AP or confocal data is retrieved from MidExDB using one of the search modes on the Home page (described in detail in Utility and discussion section). For example, when using Gene Search to find a gene (Figure 2A), the resulting Images link returns DIC images of whole-mount AP in situ hybridization gene expression data arranged by stage. The Confocal link returns a tabular summary of confocal expression data and a link to the raw data displayed as movies.

### AP data

MidExDB currently contains 6987 images acquired from in situ hybridization experiments documenting the embryonic expression patterns of 286 identified midline-expressed genes. All images in MidExDB were derived from wild-type embryos. The 286 genes include the previously-published 249 [2,3,5] and an additional 37 genes reported here (Table 1). Hybridization was visualized using a histochemical AP-NBT/BCIP reaction and examined by DIC microscopy [2]. For many genes, images are provided at each stage of development from stages 5 to 17. Midline expression in an individual embryo is often shown at multiple magnifications ranging from 12.5× to 100×. DIC imaging of AP histochemically-stained whole-mount embryos allows for analysis of midline gene expression in a large number of embryos at many stages. This is particularly useful for describing the temporal component of gene expression. However, they also provide insight into which midline cells express the gene, such as determining whether they are expressed in all midline cells or a subset of cells. In the mature CNS, it can often be determined whether the gene is expressed in MG or midline neurons based on their positions. However, it is difficult to assign with certainty exactly in which midline cell types the gene is expressed, particularly at earlier stages of development, such as stages 10-11. In this regard, confocal analysis (described under Confocal data) can determine exactly in which cell type a gene is expressed.

**Table 1:** Midline-expressed genes newly added to MidExDB.

**Symbol**	**Function**	**Symbol**	**Function**
α-Adaptin	Signaling	*Hsp27*	Chaperone
*ac*	Transcription factor	*KrT95D*	Metabolism
*CG11347*	Unknown	*Lis-1*	Cytoskeleton
*CG13248*	Transporter	*Lola*	Transcription factor
*CG14968*	Unknown	*Mid*	Transcription factor
*CG15117*	Metabolism	*Nuf*	Cytoskeleton
*CG2893*	Neural function	*Oc*	Transcription factor
*CG31088*	Unknown	*Oli*	Transcription factor
*CG6044*	Unknown	*Proct*	Neural function
*CG6847*	Metabolism	*Pvf2*	Signaling-receptor
*CG9336*	Unknown	*shaI*	Neural function
*CG9743*	Metabolism	*Slim*	Cytoskeleton
*ck*	Cytoskeleton	*Spi*	Signaling
*cry*	Circadian rhythms	*Sqz*	Transcription factor
*ct*	Transcription factor	*Sty*	Signaling
*Gp150*	Signaling	*ush*	Transcription factor
*grim*	Apoptosis	*Vmat*	Neural function-transporter
*gsb*	Transcription factor	*W*	Apoptosis
*hbs*	Adhesion		

**Figure 3:**
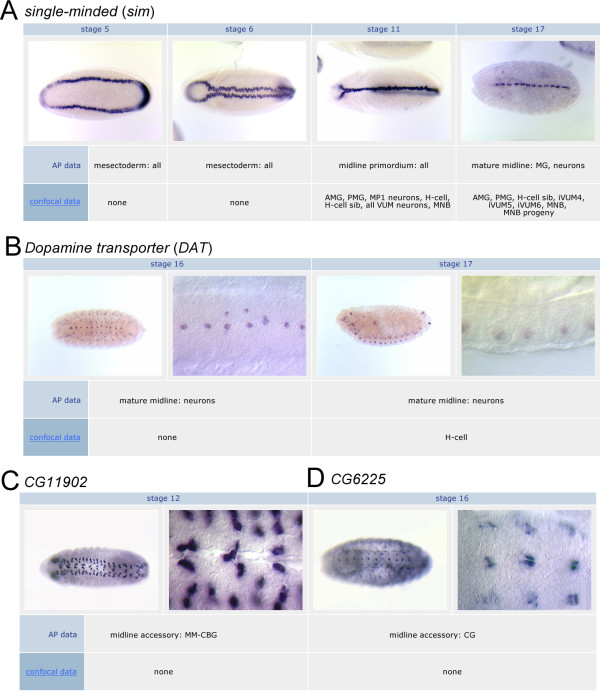
**Gene expression during midline cell development**. Representative MidExDB images from multiple stages of embryonic development are shown for (A) *sim*, (B) *DAT*, (C) *CG11902*, and (D) *CG6225*. The *sim *and *DAT *genes are expressed in midline cells derived from *sim*^+ ^mesectodermal cells, whereas *CG11902 *and *CG6225 *are expressed in midline accessory cells. *CG11902 *is expressed in medial most-cell body glia (MM-CBG) and *CG6225 *is expressed in channel glia (CG). Ventral and sagittal views are shown at either low (12.5×) or high (50×) magnification. Below each image is a description of its expression, both for the AP data and for the confocal data. Stage 16 ventral views of *DAT *show its midline localization.

To categorize midline gene expression of AP data, a controlled vocabulary containing both temporal and cell-type terms was used, and is described in detail under the "Images" Help topic located on the Help page (the controlled vocabulary only applies to the AP data and not confocal data, since the confocal data determines gene expression precisely at the single-cell level). Temporally, midline cell development is subdivided into 3 major time periods: mesectoderm (stages 5-8), midline primordium (stages 9-12), and mature midline (stages 13-17). Regarding cell type, different terms were assigned for each developmental period. For the mesectoderm and midline primordium time periods, there are only two descriptors: all midline cells or a subset. As an example, several representative images corresponding to the *sim *gene are shown (Figure 3A). The annotation indicates that *sim *is expressed in all midline cells during the mesectoderm and midline primordium periods. For the mature midline stages, gene expression was divided into three categories: midline cells, midline accessory cells, and apoptotic cells [2]. The midline cells include the midline neurons and MG that are derived from the *sim*^+ ^mesectodermal cells. For example, both the *sim *gene and the *Dopamine transporter *(*DAT*) gene are expressed in midline neurons, while *sim *is also expressed in MG (Figure 3A, B). Midline accessory cells reside at the midline, but are not derived from the *sim*^+ ^mesectodermal cells, and instead originate from either the lateral CNS or the mesoderm. These include the medial most-cell body glia (MM-CBG; Figure 3C), channel glia (CG; Figure 3D), and dorsal median cells. Apoptotic cells are dying cells that lie between the CNS and epidermis. Both temporal and cell type subcategories can be searched and compared in MidExDB.

### Confocal data

**Figure 4:**
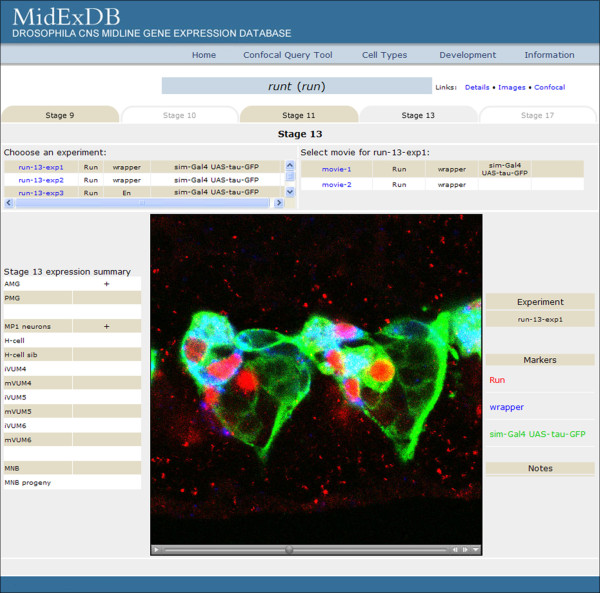
**Movies of confocal data**. Upon selecting the "Images available" link on the confocal data page (Figure 2B), a confocal experiments page opens. Selecting a tab matching a developmental stage reveals a list of experiments, each corresponding to a separate Z-series stack. The antibodies and probes employed in the confocal stack are indicated for each experiment. Selection of an experiment lists one or more movies derived from that stack with markers to the right. Selection of a movie link results in a QuickTime movie appearing in a window. The color-coded in situ probes or antibodies are listed to the right of the movie. The control bar underneath the movie allows the user to manually scroll through the movie. In the example shown, the *sim-Gal4 UAS-tau-GFP *embryo was stained for Run protein (red), *wrapper *RNA (blue), and GFP (green). The table to the left of the movie summarizes the data for each gene at the selected stage assembled from all experiments, not just the Z-series shown.

Selecting "Confocal" from Gene Search (Figure 2A) returns a dataset (Figure 2B) derived from confocal imaging of fluorescence in situ hybridization and immunostaining data [3,5]. These data have single-cell resolution, and gene expression was assigned to individual cell types by colocalization with cell type-specific markers. Five embryonic stages (9, 10, 11, 13, and 17) were selected for analysis because they correspond to periods of major midline developmental changes. The confocal data currently exists for 77 genes, and is represented by a text summary describing expression in each midline cell type at each stage examined (Figure 2B). QuickTime movies generated from the 3-dimensional stacks (Z-series) for each gene can be viewed by selecting the "Images available" link above the confocal text summary (Figure 2B). The confocal experiments page opens (Figure 4), and different experiments are listed for each stage of development. There are often multiple experiments (Z-series stacks) for each stage. Upon selecting a particular experiment, links to QuickTime movies appear and can be viewed. The in situ probes and antibodies are listed to the right of each movie. After a movie is selected and appears in the movie window, there is a control bar at the bottom of the movie that allows the choice of playing the movie or pausing on individual frames. Figure 4 shows an image that displays *runt *(*run*) expression at stage 13. In this movie, the labels depict Run protein, *wrapper *RNA, and midline cells visualized by anti-GFP staining of a *sim-Gal4 UAS-tau-GFP *embryo [3]. Anti-GFP staining of *sim-Gal4 UAS-tau-GFP *embryos allows specific visualization of all midline cells from stage 10-17, and greatly enhances midline cell identification [3]. In this movie, Run was shown to colocalize with *wrapper*. Since *wrapper *is strongly expressed in AMG and weakly in PMG at stage 13, this indicated that *run *is expressed in AMG, as indicated to the left of the movie. Additional experiments with other markers indicated that *run *is also present in midline precursor 1 (MP1) neurons. These movies are intended to provide the original data that cell type-specific expression assignments for each gene were based, as well as assist other labs interested in studying the *Drosophila *midline cells.

### Summary of midline gene expression data

The 286 midline-expressed genes can be grouped by expression and function. There are 44 genes expressed during the mesectodermal period, 162 at the midline primordium stage, and 198 in mature midline cells (these numbers do not include those genes with potential or uncertain midline expression). Of the genes expressed in mature midline cells, 54 are expressed in MG, 137 in midline neurons, 6 in both, and for 9 genes, the expression cannot be unambiguously assigned. There are 34 genes expressed in midline accessory cells, and 4 present in apoptotic cells dying at the midline. Regarding function, the largest group of genes is concerned with transcription, including 83 members. There are 50 genes listed as cell signaling, and 26 are neural function genes, which encode neurotransmitter biosynthetic enzymes, neuropeptides, membrane transporters, vesicular transporters, and neurotransmitter receptors. The remaining genes are currently partitioned into an additional 21 classes. Previously, we estimated that the 286 midline-expressed genes in this database likely represented >50% of the genes expressed in midline cells (this does not generally include broadly-expressed genes also present in midline cells) [2].

## Utility and discussion

### Search modes

MidExDB has two major search modes: basic data searches and the confocal query tool. The basic data searches return information from AP data and confocal data, while the confocal query tool returns information based on expression in specific cell types. For convenience, the basic data searches (Category, Gene, and Quick) are located on the MidExDB Home page, and the confocal query tool can be accessed on the navigation menu (Figure 2A). Under Category Search, the user can choose to list genes based on: (1) Gene Data (name, symbol or short name, CG#, protein type, or function), (2) Expression Data (AP data), and (3) Confocal Data. Each category is divided into subcategories. For example, the "Gene Data -Midline cell" category can be searched for expression in mesectoderm, midline primordium, mature midline, midline neurons, or MG. The Gene Data category selection results in a list of all genes that is sorted based on selection of a subcategory entry, such as name, protein type, or function. If protein type or function is chosen, then another menu provides a list of proteins and functions to select. For example, selection of "Protein Type" and "bHLH-PAS transcription factor" returns two entries: *cycle *and *sim*. Once the list is present, it can be viewed in multiple ways to highlight different aspects of the gene's features and expression. The data is also available in a printable format that can be copied into a database or spreadsheet.

Using Gene Search, the name of a gene is entered and three views are returned: Details, Images, and Confocal. It is also possible to analyze multiple genes together using the Batch Search. The Details view provides an overview of data for that gene. This includes protein type and function, midline cell and midline accessory cell expression data determined from AP data, and an indication if confocal data is available with a link to access that information. There are also links to the FlyBase [6] and the BDGP gene expression database [4] entries for the gene. The Images view displays AP histochemically-stained images of midline gene expression from embryonic stages 5 to 17. Below the row of images for each stage is a description of the expression pattern using the controlled vocabulary (Figure 3A-D). The descriptions for stages 9, 10, 11, 13, and 17 also include the results of confocal analysis for those genes analyzed. As an example, an entry for *sim *is shown for stage 17 in Figure 3A, and *DAT *at stage 17 in Figure 3B. They are both annotated as expressed in midline neurons based on the AP data, whereas the confocal data indicates that *DAT *is expressed in one neuron (H-cell), while *sim *is expressed in a larger set of midline neurons (H-cell sib, ventral unpaired median interneurons 4-6 (iVUMs4-6), median neuroblast progeny (MNB progeny), as well as the median neuroblast (MNB) and AMG. The Confocal view (Figure 2B) provides a text-based summary of confocal expression data for the selected gene. The confocal data is divided into stages 9, 10, 11, 13, 17 with each corresponding cell type listed.

At the bottom of the MidExDB home page are 6 Quick Searches for AP data or confocal data (Figure 2A). The quick searches can be used to retrieve a list of all genes with either AP data or confocal data. There is also a quick search function for retrieving all genes expressed in MG or midline neurons. From the retrieved lists, clicking on the gene symbol will open the Details page for that gene.

The second major search mode, the Confocal Query Tool allows the user to customize a query to identify genes expressed in specific cell types at defined developmental stages (Figure 2C). Each cell type is listed for each of the 5 developmental stages in which confocal data is present. Complex queries using the Boolean operators AND, OR, and NOT are possible. As an example (Figure 2C), the database can be searched for genes expressed at: stage 13 in the MP1 neurons AND H-cell sib but NOT H-cell. Only 1 gene, *fork head *(*fkh*) appears. Its expression at each stage of development is listed in tabular form (Figure 2C). Lists of genes obtained using the Confocal Query Tool can be printed or exported to the Category Search to view additional data (for example, protein type) for each gene in the list.

### Descriptive background information and protocols

**Figure 5:**
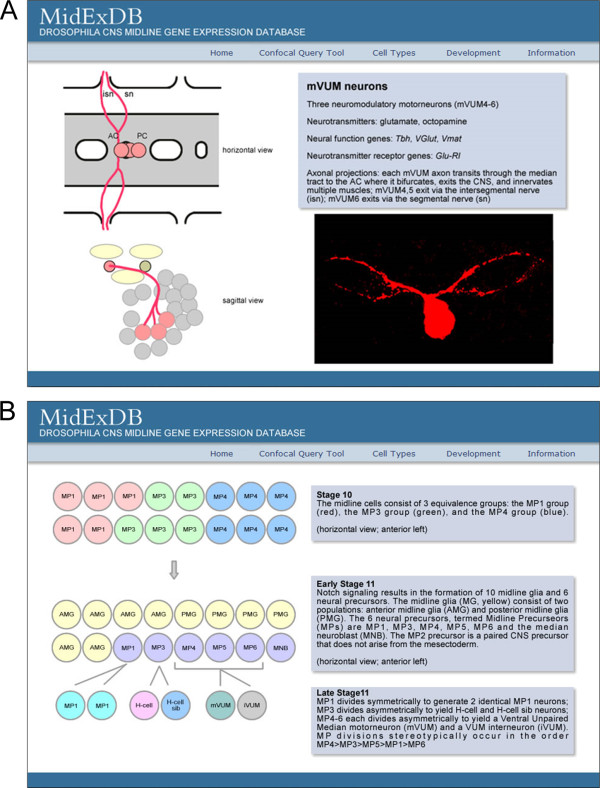
**Descriptions of midline cell types and development**. (A) The Cell Types menu allows selection of information regarding each midline cell type. Shown is a representative webpage summarizing the ventral unpaired median motorneurons (mVUM neurons). (B) The Development menu has a selection of different developmental stages. Shown are schematics and commentaries describing midline cell development at stages 10 and 11.

Two types of background information on midline cells are provided in the navigation bar. The first, Cell Types, is a description of all of the midline cell types, including MG, midline neurons, and midline accessory cells (Figure 5A). The summary of each neuronal type includes schematic drawings illustrating cell position and axonal trajectories; in addition, a confocal image showing the axonal trajectories is included. The text description includes information regarding the neurotransmitters and neurotransmitter receptors present in the cell, as well as a list of genes expressed in the mature neurons that contribute to their neural properties. The second set of information is Development, in which midline cell development is presented as a series of schematics from stages 5-17 that are accompanied by commentary (Figure 5B). Key references to support this model are included in the References selection under the Information tab.

The Protocols section in the Information menu provides PDFs of detailed experimental protocols that were used to generate the data present in MidExDB. Antibody staining and in situ hybridization protocols are described for both fluorescence and AP detection, including the use of Tyramide Signal Amplification (TSA).

## Conclusion

The goal of MidExDB is to disseminate information about the *Drosophila *CNS midline cells. MidExDB is able to search and integrate a large-scale midline gene expression dataset. The database provides a useful foundation for studying CNS midline development and function, particularly the underlying regulatory circuitry.

## Availability and requirements

MidExDB is accessible at  or via the Crews Lab homepage at . Any modern web browser including Mozilla Firefox, Safari, or Microsoft Internet Explorer is sufficient to access the database.

## Authors' contributions

SRW designed and implemented the database scheme and the web pages. SBS participated in the design of the web pages and their content. STC conceived the database and participated in its design and coordination. All authors read and approved the manuscript.

## References

[B1] Jacobs JR (2000). The midline glia of Drosophila: a molecular genetic model for the developmental functions of glia. Prog Neurobiol.

[B2] Kearney JB, Wheeler SR, Estes P, Parente B, Crews ST (2004). Gene expression profiling of the developing Drosophila CNS midline cells. Dev Biol.

[B3] Wheeler SR, Kearney JB, Guardiola AR, Crews ST (2006). Single-cell mapping of neural and glial gene expression in the developing *Drosophila *CNS midline cells. Dev Biol.

[B4] Tomancak P, Beaton A, Weiszmann R, Kwan E, Shu S, Lewis SE, Richards S, Ashburner M, Hartenstein V, Celniker SE, Rubin GM (2002). Systematic determination of patterns of gene expression during Drosophila embryogenesis. Genome Biol.

[B5] Wheeler SR, Stagg SB, Crews ST (2008). Multiple Notch signaling events control Drosophila CNS midline neurogenesis, gliogenesis and neuronal identity. Development.

[B6] Tweedie S, Ashburner M, Falls K, Leyland P, McQuilton P, Marygold S, Millburn G, Osumi-Sutherland D, Schroeder A, Seal R, Zhang H, FlyBase Consortium (2009). FlyBase: enhancing Drosophila Gene Ontology annotations. Nucleic Acids Res.

